# Influence of *Ascophyllum nodosum* Extract Foliar Spray on the Physiological and Biochemical Attributes of Okra under Drought Stress

**DOI:** 10.3390/plants11060790

**Published:** 2022-03-16

**Authors:** Jawad Ali, Ibadullah Jan, Hidayat Ullah, Nazeer Ahmed, Mukhtar Alam, Rafi Ullah, Mohamed El-Sharnouby, Hosny Kesba, Mustafa Shukry, Samy Sayed, Taufiq Nawaz

**Affiliations:** 1 Department of Agriculture, University of Swabi, Swabi 23561, Pakistan; jwd.ali88@gmail.com (J.A.); dribad@uoswabi.edu.pk (I.J.); drhidayat@uoswabi.edu.pk (H.U.); mukhtar@uoswabi.edu.pk (M.A.); rafiullah@uoswabi.edu.pk (R.U.); 2 Department of Biotechnology, College of Science, Taif University, P.O. Box 11099, Taif 21944, Saudi Arabia; m.sharnouby@tu.edu.sa; 3 Zoology and Agricultural Nematology Department, Faculty of Agriculture, Cairo University, Giza 12613, Egypt; hosny.hosny@agr.cu.edu.eg; 4 Department of Physiology, Faculty of Veterinary Medicine, Kafrelsheikh University, Kafrelsheikh 33516, Egypt; mostafa.ataa@vet.kfs.edu.eg; 5 Department of Science and Technology, University College-Ranyah, Taif University, P.O. Box 11099, Taif 21944, Saudi Arabia; 6 Department of Food Science and Technology, The University of Agriculture, Peshawar 25130, Pakistan; taufiqnawaz85@gmail.com

**Keywords:** seaweed, *Abelmoschus esculentus* L., chlorophyll content, antioxidant enzymes, phenolic compounds, abiotic stress

## Abstract

Drought stress restricts the growth of okra (*Abelmoschus esculentus* L.) primarily by disrupting its physiological and biochemical functions. This study evaluated the role of *Ascophyllum nodosum* extract (ANE) in improving the drought tolerance of okra. Drought stress (3 days (control), 6 days (mild stress), and 9 days (severe stress)) and 4 doses of ANE (0, 0.1%, 0.2%, and 0.3%) were imposed after 30 days of cultivation. The results indicate that drought stress decreases the chlorophyll content (total chlorophyll, chlorophyll a, chlorophyll b, and carotenoid) but increases the activity of anthocyanin, proline, and antioxidant enzymes such as ascorbate peroxidase (APX), peroxidase (POD), and catalase (CAT). Physiological and biochemical plant disturbances and visible growth reduction in okra under drought stress were significantly decreased by the application of ANE foliar spray. ANE spray (0.3%) significantly increased the chlorophyll abundance and activity of anthocyanin, proline, and antioxidants (APX, POD, and CAT). ANE regulated and improved biochemical and physiological functions in okra under both drought and control conditions. The results of the current study show that ANE foliar spray may improve the growth performance of okra and result in the development of drought tolerance in okra.

## 1. Introduction

Climate change has become a major threat worldwide and, consequently, is a subject of significant research focus. This has created a large problem for the economic growth of many countries [1]. Water limitation is a severe risk for the food safety of affected countries, and climate change has increased the occurrence of risky events such as drought [2]. Under drought stress, the presence of active forms of oxygen leads to oxidative stress, which damages many cellular constituents such as carbohydrates, lipids, nucleic acids, and proteins, ultimately retarding plant growth, respiration, and photosynthesis [3,4]. *Ascophyllum nodosum* extract has become an element of interest to many biologists due to its physiological and toxicological importance. It plays a vital role in plant growth under conditions of abiotic stress, enhancing photosynthetic pigments, phenolic compounds, and antioxidant activity, and improving plant tolerance to water deficit conditions by regulating their water status [5]. Unfavorable conditions often damage plants, retarding their growth and yield [6]. Concerning adverse conditions, drought plays a vital role in decreasing crop production worldwide [7]. Drought restricts the genetic potential of a plant species during its life cycle. Drought stress damages drought-sensitive plants at the vegetative level and limits the reproductive development of drought-resistant plants [8].

Stomata closure is the initial response to drought, and this inhibits physiological and biochemical responses in plants [4]. Moreover, this process alters the developmental process by driving plants to adopt a defensive state where plant productivity is hindered in the long term [9].

The generation of reactive oxygen species and active oxygen forms under drought stress causes surplus oxidative excitation energy, which is responsible for the destruction of photosystems due to the low regulation of photosynthetic carbon metabolism genes [10]. The occurrence of an appropriate response to stomatic and nonstomatic restriction in crop photosynthesis depends on the intensity of the drought stress and the tendency of a plant to dry [11].

Plants adapt and adjust to drought condition by protein and osmolyte accumulation [12]. Accumulated osmolytes and proteins behave as osmotic readjustment promoters, improving the osmotic equilibrium and reducing cell injury under conditions of water scarcity [13]. Osmolytes consist of amino acids such as proline that help with the adaptation to drought conditions [14]. In addition, morphological characteristics, such as root thickness, depth, mass, and development characteristics, such as the ability of the roots to disrupt the compact layer of soil, minimize stress resistance [15].

The evolution of highly resistant genotypes using breeding approaches is required to enhance the productive capacity of plants under water-deficient circumstances [16]. Additionally, the exogenous application of various growth promoters has been estimated across the globe to boost drought tolerance [4]. However, the use of such attributes is usually costly. Recently, the use of mineral supplements for stress resistance has received significant attention [17].

Extracts of brown seaweeds are largely used to reduce the harmful effects of abiotic stress and enhance plant growth. The chemical composition of these seaweeds is mainly fatty acids, phytohormones, vitamins, mineral nutrients, and polysaccharides. Research has investigated the probable molecular machinery influenced by seaweed extracts (SWEs). Brown SWEs are composed of different types of organic and inorganic components. The inorganic components of *Ascophyllum nodosum* extract (ANE) are nitrogen, phosphorus, potassium, calcium, iron, magnesium, zinc, sodium, and sulfur. Various organic compounds contain osmolytes that are found in ANE. Brown seaweed has been shown to contain phytohormones—inorganic and organic components that act against the harshness of drought stress and enhance crop production and yield [18]. *Ascophyllum nodosum* is one of the most widely used seaweeds in agriculture. It is a brown seaweed that is found only in the North Atlantic Ocean [19]. *Ascophyllum nodosum* is frequently applied in agriculture, especially as an extract. In previous studies, ANE was shown to enhanced freezing and salinity tolerance in plants [20,21].

Okra (*Abelmoschus esculentus* L.) belongs to the Malvaceae family and is very popular in the subcontinent. It is native to Ethiopia and Sudan while ranking number one in India in terms of cultivation. In many countries, it is a historically important medicinal crop. It has been bred in Africa, America, Asia, and Europe [22]. Okra has played a vital role in mitigating food insecurity and malnutrition in developing countries. Its use as an indigenous vegetable is being promoted for this purpose. However, in the past, it was considered a minor crop, and no care was taken to improve it at an international level using research programs [23]. On the other side, the demand for vegetable oil is increasing rapidly with the increase in the human population and because of the industrial revolution regarding the health-promoting components in oils. Because of the presence of some underutilized and newer resources, vegetable oil is a subject of concern [24]. Okra seeds contain 20–40% oil; therefore, there is potential to grow okra as an oil seed crop as well as vegetable crop [25]. Okra is susceptible to abiotic stresses, such as drought stress, frost, chilling temperature, and water-logged conditions. The cultivars have adopted certain behaviors based on the climate of the country in which they grow [26].

Drought negatively affects plant morphological and physiological traits, reduces the leaf water potential and sap movement, and changes the plant water status [27]. The okra plant is usually grown for its green tender pods in arid and semiarid regions. The duration and number of irrigation cycles (irrigation interval) required affect the quality of the pod yield. Water deficit in okra leads to a poor yield. The yield reduction depends on the magnitude of water deficit and the stage of plant growth at the time that drought stress occurs. A maximal reduction has been shown to occur when water stress occurs at the flowering and pod formation stages, because these stages are the most sensitive to drought [28]. Water stress at the beginning of flowering increases embryo abortion, while water stress after flowering drastically affects the yield [29]. Significant differences among okra genotypes regarding the plant height, number of days to flowering, number of branches per plant, fruit length, fruit weight and size, early yield, and total yield have been observed under drought conditions. In all cultivars, the maximum yield reduction in okra plants occurs when drought stress takes place at the flowering and pod-filling stages, while the effects of drought at the vegetative stage are not as severe [30]. Among the growth stages of okra, water stress at the reproductive stage has been reported as the most destructive for the yield of plants growing in arid and semiarid regions [31].

Much work has been conducted on the elimination of the harmful effects of abiotic stress through the application of biofertilizer, but little or no literature is available on the effects of ANE on the physiological and biochemical attributes of okra under drought stress conditions. The current study evaluated the effects of ANE on the physiological and biochemical attributes of okra plants under drought stress. This included measures of chlorophyll, carotenoids, anthocyanin, proline, and antioxidant enzyme activity.

## 2. Results

### 2.1. Chlorophyll-a Content (µg mL^−1^)

**Figure 1:**
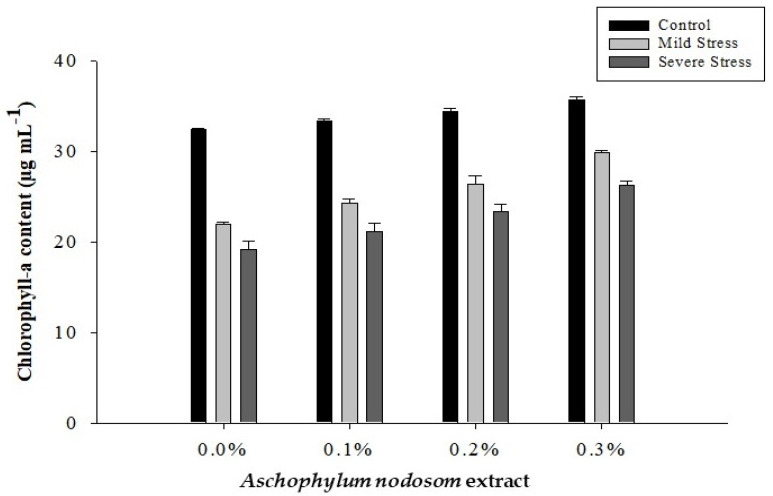
Chlorophyll-a content of okra. The figure shows the effect of the interaction of *Ascophylum nodosum* extract and drought stress.

It is clear from Figure 1 and Table 1 that the chlorophyll-a content in the susceptible okra cultivar “Super Green” was significantly affected by drought conditions, ANE levels, and their interaction.

The maximum (34.01 µg mL^−1^) chlorophyll-a content was observed in control plants, while the minimum chlorophyll-a (22.50 µg mL^−1^) content was observed in plants subjected to different drought conditions. In contrast, exogenous application of ANE significantly increased the chlorophyll-a content under both drought and control conditions. A high chlorophyll-a (30.61 µg mL^−1^) content was obtained in plants treated with a high dose of ANE (0.3%), while the lowest chlorophyll-a (24.56 µg mL^−1^) content was noted in control plants. Similarly, regarding the interaction between ANE application and drought stress, the maximum chlorophyll-a (35.69 µg mL^−1^) content was recorded in plants treated with 0.3% ANE under control conditions. In comparison, the lowest chlorophyll-a (19.19 µg mL^−1^) content was noted in plants subjected to different drought stresses and no application of ANE (Figure 1).

### 2.2. Chlorophyll-b Content (µg mL^−1^)

**Figure 2:**
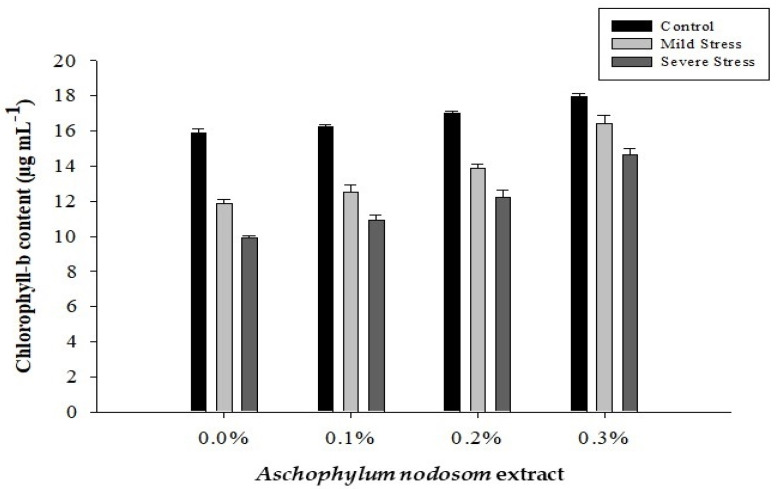
The effects of *Ascophylum nodosum* extract and drought stress conditions on the chlorophyll-b content of okra.

The chlorophyll-b content of okra leaves was significantly affected by drought conditions, ANE levels, and their interaction, as shown in Table 1 and Figure 2.

The maximum chlorophyll-b (16.77 µg mL^−1^) content was observed in well-watered plants, while the minimum chlorophyll-b (11.92 µg mL^−1^) content was recorded in plants subjected to different drought conditions. Moreover, exogenous application of ANE significantly enhanced the chlorophyll-b content under drought and well-watered conditions. A high chlorophyll-b (16.35 µg mL^−1^) content was obtained in plants exposed to a high dose of ANE (0.3%), while the lowest chlorophyll-b (12.55 µg mL^−1^) content was recorded in plants that were not treated with ANE. Similarly, the interactive effect of ANE and drought levels had a significant effect on the chlorophyll-b content. The maximum chlorophyll-b (17.95 µg mL^−1^) content was recorded in plants treated with 0.3% ANE under control conditions, while the lowest chlorophyll-b (9.94 µg mL^−1^) content was obtained in plants subjected to different drought stresses and no ANE (Figure 2).

### 2.3. Total Chlorophyll Content (µg mL^−1^)

**Figure 3:**
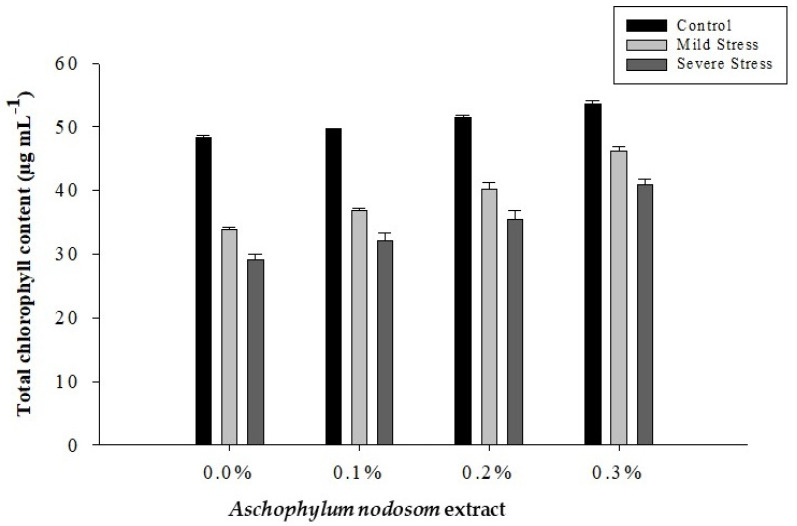
The effects of *Ascophylum nodosum* extract and drought stress conditions on the total chlorophyll content of okra.

The total chlorophyll content of okra leaves was significantly affected by drought conditions, ANE levels, and their interaction, as shown in Table 1 and Figure 3.

The maximum total chlorophyll content (50.78 µg mL^−1^) was observed in plants under no stress, while the minimum total chlorophyll content (34.42 µg mL^−1^) was observed in plants subjected to different drought conditions. Additionally, the exogenous application of ANE significantly enhanced the total chlorophyll content under drought and well-watered conditions. A high total chlorophyll content (46.96 µg mL^−1^) was recorded in plants sprayed with 3% ANE, while the lowest total chlorophyll content (37.11 µg mL^−1^) was observed in control plants. Similarly, regarding the interaction between ANE application and drought stress, the maximum total chlorophyll content (53.65 µg mL^−1^) was recorded in plants treated with 0.3% ANE under control conditions; however, the lowest total chlorophyll content (29.12 µg mL^−1^) was obtained in plants subjected to different drought conditions and no ANE (Figure 3).

### 2.4. Carotenoid Content (µg mL^−1^)

**Figure 4:**
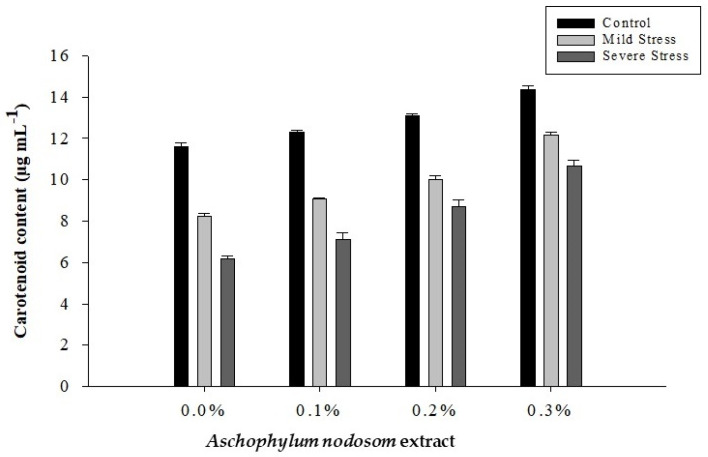
The effects of *Ascophylum nodosum* extract and drought stress conditions on the carotenoid content of okra.

The carotenoid content of okra leaves was significantly affected by drought conditions, ANE levels, and their interaction, as shown in Table 1 and Figure 4.

The maximum carotenoid (12.85 µg mL^−1^) content was observed in control plants, while the minimum carotenoid (8.17 µg mL^−1^) content was observed in plants subjected to different drought conditions. Additionally, the exogenous application of ANE significantly enhanced the carotenoid content of okra plants under various drought levels. A high carotenoid (12.40 µg mL^−1^) content was recorded in plants sprayed with 0.3% ANE, while the lowest carotenoid (8.67 µg mL^−1^) content was recorded in plants that were not treated with ANE. Similarly, regarding the interaction between ANE application and drought stress, the maximum carotenoid (14.37 µg mL^−1^) content was recorded in plants treated with 0.3% ANE under control conditions. In comparison, the lowest carotenoid (6.19 µg mL^−1^) content was observed in plants subjected to severe stress and no ANE (Figure 4).

### 2.5. Anthocyanin Content (mg g^−1^)

**Figure 5:**
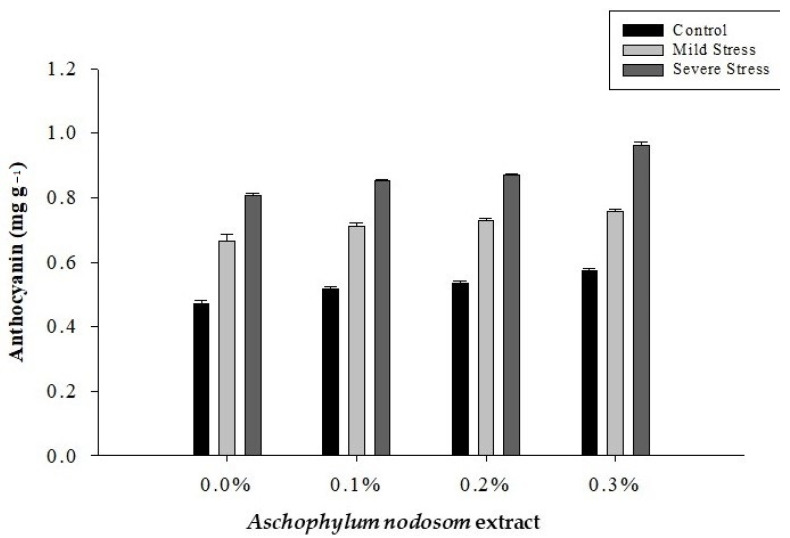
The effects of *Ascophylum nodosum* extract and drought stress conditions on the anthocyanin content of okra.

The data in Table 1 regarding the mean anthocyanin content reveal a significant variation in the anthocyanin content of the leaves of the susceptible okra cultivar “Super Green” depending on the drought conditions, ANE levels, and their interaction (Figure 5).

The maximum anthocyanin (0.8728 mg g^−1^) content was recorded in plants under severe stress, while the lowest anthocyanin (0.5235 mg g^−1^) content was observed in control plants. Similarly, the highest anthocyanin (0.7635 mg g^−1^) content was noted in plants treated with 0.3% ANE, whereas the minimum anthocyanin (0.6479 mg g^−1^) content was recorded in untreated plants. Regarding the interaction between drought stress and ANE application, the highest anthocyanin (0.9606 mg g^−1^) content was observed in plants treated with 0.3% ANE under severe drought stress conditions. In comparison, the lowest anthocyanin (0.4706 mg g^−1^) content was observed in control plants (Figure 5).

### 2.6. Proline Content (µmole g^−1^)

**Figure 6:**
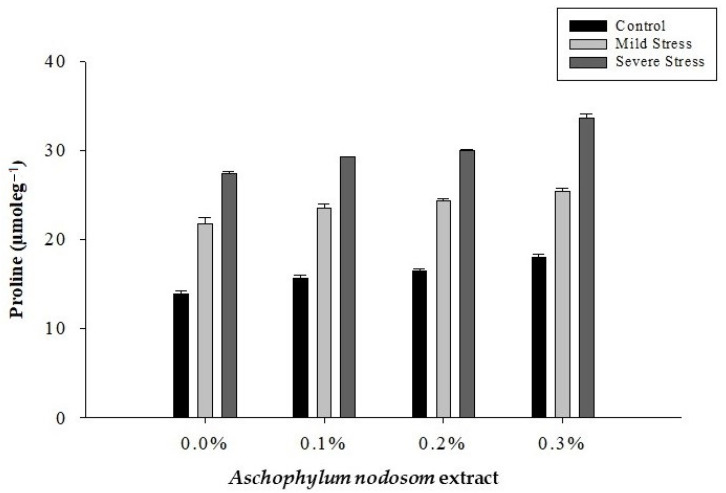
Proline accumulation in okra and the effects of *Ascophylum nodosum* extract and drought stress.

The proline content of the leaves of the susceptible okra cultivar “Super Green” was significantly affected by the drought conditions, ANE level, and their interaction, as shown in Table 2 and Figure 6.

The maximum proline (30.06 µmole g^−1^) content was recorded in plants subjected to severe stress, while the lowest proline (15.98 µmole g^−1^) content was observed in well-watered plants. Similarly, the highest proline (25.65 µmole g^−1^) content was noted in plants treated with 0.3% ANE, whereas the minimum proline (20.99 µmole g^−1^) content was recorded in plants not treated with ANE. Regarding the interaction between drought stress and ANE application, the maximum proline (33.59 µmole g^−1^) content was recorded in plants treated with 0.3% ANE under severe stress conditions, whereas well-watered plants not treated with ANE produced less (13.85 µmole g^−1^) proline (Figure 6).

### 2.7. Ascorbate Peroxidase Activity (APX) (µ mg^−1^ Protein)

**Figure 7:**
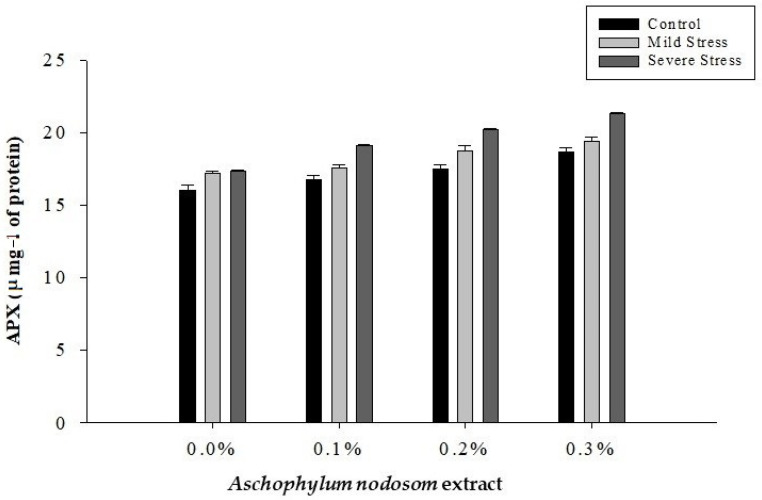
The effects of *Ascophylum nodosum* extract and drought stress conditions on the APX activity of okra.

The APX activity in the leaves of the susceptible okra cultivar “Super Green” was significantly affected by drought conditions, ANE levels, and their interaction, as shown in Table 2 and Figure 7.

The maximum level of APX (19.49 µ mg^−1^ protein) activity was recorded in plants subjected to severe stress, while the lowest level of APX (17.24 µ mg^−1^ protein) activity was observed in well-watered plants. Similarly, the highest level of APX (19.79 µ mg^−1^ protein) activity was observed in plants treated with 0.3% ANE, whereas the lowest APX activity (16.87 µ mg^−1^ protein) was recorded in plants not treated with ANE. Regarding the interaction between ANE application and drought stress, the maximum APX (21.33 µ mg^−1^ protein) activity was recorded in plants treated with 0.3% ANE under severe stress, whereas well-watered plants not treated with ANE produced less APX (16.05 µ mg^−1^ protein) activity (Figure 7).

### 2.8. Peroxidase Activity (POD) (µ mg^−1^ Protein)

**Figure 8:**
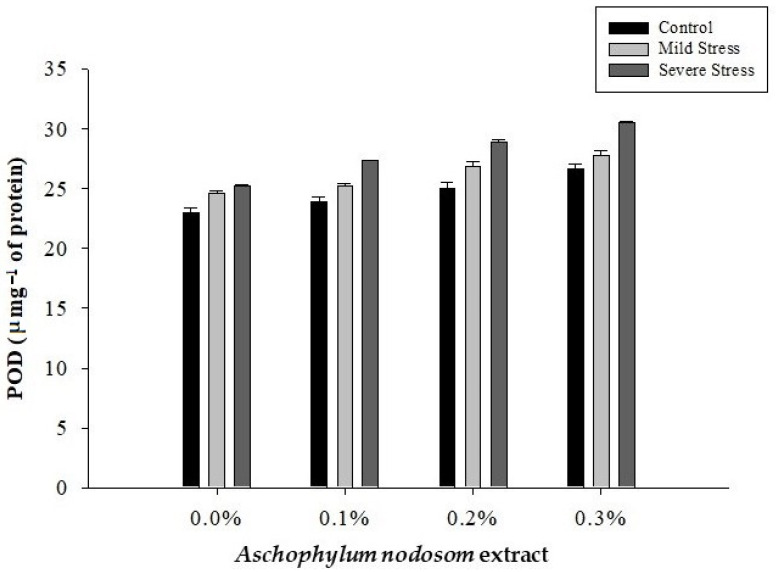
The effects of *Ascophylum nodosum* extract and drought stress conditions on the POD activity of okra.

The POD activity of the leaves of the susceptible okra cultivar “Super Green” was significantly affected by drought conditions, ANE levels, and their interaction, as shown in Table 2 and Figure 8.

The maximum level of POD (28.01 µ mg^−1^ protein) activity was recorded in plants subjected to severe stress, while the lowest level of POD (24.67 µ mg^−1^ protein) activity was observed in well-watered plants. Similarly, the highest level of POD (28.34 µ mg^−1^ protein) activity was noted in plants treated with 0.3% ANE, whereas the minimum POD activity (24.29 µ mg^−1^ protein) was recorded in plants not treated with ANE. In terms of the interaction between drought stress and ANE application, the maximum level of POD (30.53 µ mg^−1^ protein) activity was recorded in plants treated with 0.3% ANE under severe stress conditions. In contrast, the lowest level of POD (22.97 µ mg^−1^ protein) activity was observed in untreated control plants (Figure 8).

### 2.9. Catalase (CAT) (µ mg^−1^ Protein)

**Figure 9:**
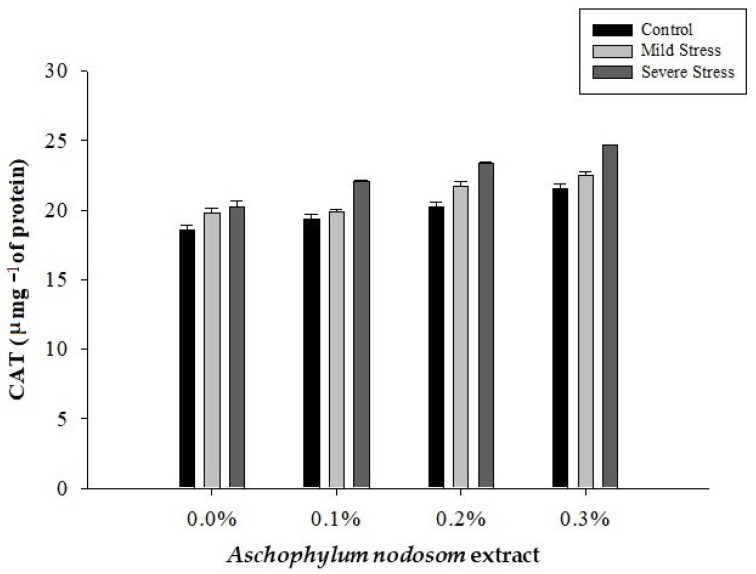
The effects of *Ascophylum nodosum* extract and drought stress conditions on the CAT activity of okra.

The activity level of catalase in the leaves of the susceptible okra cultivar “Super Green” was significantly affected by drought conditions, ANE levels, and their interaction, as shown in Table 2 and Figure 9.

The maximum level of CAT (22.58 µ mg^−1^ protein) activity was recorded in plants grown subjected to severe stress, while the lowest level of CAT (19.93 µ mg^−1^ protein) activity was observed in well-watered plants. Similarly, the highest level of CAT (22.89 µ mg^−1^ protein) activity was noted in plants treated with 0.3% ANE, whereas the minimum level of CAT (19.52 µ mg^−1^ protein) activity was recorded in plants grown without ANE. Regarding the interaction between drought stress and ANE application, the maximum level of CAT (24.66 µ mg^−1^ protein) activity was recorded in plants treated with 0.3% ANE under severe stress conditions, whereas well-watered plants not treated with ANE had the lowest levels of CAT (18.56 µ mg^−1^ protein) activity (Figure 9).

## 3. Discussion

Seaweed extracts play a positive role in enhancing the effectiveness of phytohormones, betaines, polymers, and nutrients [32]. ANE increases the chlorophyll content by upregulating the genes involved in photosynthesis, cell metabolism, stress response, and S and N metabolism, and inhibits senescence by downregulating cysteine proteases [33]. Seaweed (*Ascophylum nodosum* extract) is a source of cytokinins, which enhance endogenous synthesis [34]. Cytokinins have protective effects on chloroplasts [34], and consequently, they affect the chlorophyll content. A considerable increase in the chlorophyll content occurs after treatment with biostimulants (*Aschophylum nodosum* extract) [35,36,37]. Accordingly, the present study found that the mean chlorophyll content was significantly higher after the application of ANE. Similar findings were reported by Longstreth et al. [38]. Kumar [39] also observed rises in chlorophyll (a, b, and total) levels in tomato crops subjected to drought and exposed to ANE. Physiological performance, especially regarding Pn and Gs, increases as the leaf thickness increases, and this is influenced by the increased chlorophyll content, which helps the leaves to better capture light [40]. With a higher amount of light due to more Chl, there is a higher possibility of producing Pn, because of the conversation of light energy into chemical energy [41]. Allen et al. [42] found that ANE enhances the production of photosynthetic pigments in tomato leaves. Further, the same results were noted by Basavaraja et al. [43] and El-Kaoaua et al. [44] in maize and *Salvia officinalis*. ANE was shown to replenish the chlorophyll and carotenoid contents when plants were treated under drought stress [45,46]. Similar chlorophyll content trends were also found in broccoli treated with *Ascophylum nodosum* extract at rate 300 L ha^−1^ by Lola-Luz et al. [47] and Goni et al. [48] in tomato crops exposed to 0.33% ANE. Oxidative stress reduces the chlorophyll and carotenoid contents [49] due to changing consumed oxygen to ROS which retards photosynthetic growth [50]. In the current experiment, the carotenoid content of okra plants subjected to drought stress was significantly recovered by applying ANE. ANE decreases ROS activity and improves the photosynthetic machinery [51]. The chlorophyll and carotenoid contents were replenished in maize plants treated with 10% ANE under drought conditions [43]. ANE enhanced the carotenoid content in *Zea mays* and *Phaseolus mungo* [41] and *Salvia officinalis* [42] by enhancing the chloroplasts in harsh environments. Anthocyanins protect plant chloroplasts from photoinhibition and photodamage during conditions of abiotic stress [50]. Anthocyanins minimize the harmful effects of abiotic stress [52]. Spinelli et al. [53] reported that anthocyanins protect cells from oxidative damage treated with 10 mL of ANE for strawberry under salinity stress, a finding that is also supported by Alam et al. [54], who found a rise in anthocyanin level of strawberry treated with ANE at a rate of 2 g L^−1^ and reported that anthocyanin reduces the harmful effects of abiotic stress and acts as a scavenger of superoxide radicals. Roussos et al. [55] reported that anthocyanins could maintain the water potential in strawberry. An increase in anthocyanins might be due to osmotic stress or an increase in plant hormone activity. The induction of secondary metabolism is one of the defense mechanisms adopted by plants to counter the effects of a saline environment [56]. Proline is one of the main electrolytes to accumulate during biotic and abiotic stresses in tomato [57]. Higher proline levels can mitigate salinity stress by stabilizing subcellular structures and scavenging free radicals while also buffering the cellular redox potential reported in crops such as purslane [58,59]. The proline level increases with increased stress and can overcome harmful effects [58]. Previous studies recorded a boost in proline accumulation in ANE-treated plants subject to drought, which might be due to the lipophilic components (LPC) present in ANE, as these may increase the proline content and this increase has been associated with an increased expression of proline synthesis genes [60]. Renuka and Rathinavel [61] reported a positive role of seaweed extracts at rate 10% in enhancing proline accumulation and overcoming drastic effects on cluster bean. Proline accumulation was also observed in tomatoes subjected to ANE application under saline conditions [39]. Goni et al. [48] and Carvalho et al. [62] demonstrated a rise in the proline level of tomato plants subjected to drought stress following ANE application. ANE-treated plants accumulate greater proline tissue levels under saline conditions [60]. Naturally, there is a balance between antioxidant enzymes and reactive oxygen species (ROS) in a system. Any stress can disturb this balance, which increases the ROS content and causes damage. Antioxidant enzyme levels increased with application of ANE, which overcame ROS damage and restored cellular homeostasis observed in salam turf grass treated with ANE at a rate of 7 mL L^−1^, reported by [63,64]. Abiotic stress, especially environmental stress (i.e., drought), leads to the production of ROS in plants, and plants produce antioxidants, flavonoids, and secondary metabolites to detoxify these ROS and protect them from abnormal conditions (i.e., stress) through protein and amino acid stabilization [65,66]. With an increase in the ROS level, the levels of antioxidant enzymes such as APX, SOD, POD, and MDA increase to overcome the harmful effects of ROS [67]. In the current study, the application of ANE induced a partial stomatal closure, associated with changes in the expression levels of genes involved in ABA-responsive and antioxidant system in response to drought stress to remove reactive oxygen species and increase the drought tolerance of the plants The preactivation of these pathways results in a stronger ability of ANE-treated plants to maintain better photosynthetic performance in *Arabidopsis* treated with ANE at rate 3 g L^−1^ in hydroponic medium [68]. In the current study, seaweed extract increased the APX activity in okra plants subjected to drought stress. This is in agreement with the findings of Zhang et al. [69], Battacharyya et al. [18], and Khan et al. [70]. ANE has been shown to increase the activity of antioxidants such as APX in plants under abiotic stress conditions [33]. POD synthesis was shown to increase with the application of ANE in tomato plants under abiotic stress [71]. The enhancement of APX and POD activity in plants subjected to abiotic stress following the application of ANE spray was demonstrated by Shukla et al. [72], who observed increases in these parameters in crops subjected to salinity stress with ANE application. Craigie [73] found that the application of ANE increased the activity of antioxidants such as POD, CAT, and APX in various plants subjected to abiotic stress. In plants, there is a balance between antioxidant enzymes and ROS that drastically changes, hampering plant growth in stressful environments and decreasing plant homeostasis; decline in ROS activity was noted in plants treated with ANE at rate 0.2% [74]. The levels of antioxidant enzymes increase under conditions of abiotic stress to return plants to homeostasis [75]. Antioxidants such as APX, SOD, POD, and MDA reduce the effects of ROS [64]. In the current experiment, CAT activity was enhanced with foliar spray of ANE, which agrees with the results of Fike et al. [76], who reported a rise in CAT activity under conditions of abiotic stress in tall fescue. A reduction in ROS following the foliar application of ANE in okra was reported by Papenfus et al. [77]. Data recorded on control plants in the current investigation are different from each other, which could be due to plant selection or preferences for specific nutrients, ions in the same environmental conditions, and physicochemical characteristics of the soil such as pH, SOM, and redox conditions reported by [78,79,80]. The variation in soil characteristics such as EC, pH, Fe/Mn ratio, SOM, texture, and bulk density may lead to conflicting results in plants under the same environment for the same species [81,82]. The variation in observed data in control plants may also be due to other soil properties posing a negative effect on plant uptake [83]. These may include (i) soil alkalinity and sorption potential of organic matter, (ii) HMs speciation, (iii) the antagonistic interaction of nutrients with each other in soils, and (iv) soil aging [84]. Other reasons could be plant species/variety [85] or cell walls of bacteria acts as binding sites to absorb nutrients or ions from soil solution [86].

## 4. Materials and Methods

An okra cultivar, “Super Green”, was sown in a pot at the Agricultural Research Station in Swabi (34.1257° N, 72.5063° E) Khyber Pakhtunkhwa, Pakistan, under a plastic tunnel. The cultivar seeds were bought from Bayer Crop Science Pakistan (Pvt.) Ltd. Pots were filled with equal amounts of garden soil and FYM (1:1). Pots (16 inches diameter and 26 inches height) were of uniform size. The experiment was replicated three times, and there were 16 plants in each treatment group. Ten okra seeds were sown in each pot during spring. Thinning was carried out by uprooting stunted and unhealthy plants while leaving four healthy and uniform okra plants/pot when plants produced true leaves. The pots were placed randomly to meet statistical requirements and eliminate any potential positioning effect. During the experiment, the mean air temperature and relative humidity were 29 °C (maximum air temperature: 32.7 °C, minimum air temperature: 22.3 °C) and 70%, respectively. A recommended dose of N–P–K was applied at the appropriate time. Drought stress was applied after 30 days of seed sowing by subjecting the okra plants to various irrigation intervals (3, 6, and 9 days). Plants irrigated after three days were considered to be control plants. Plants irrigated at 6 days were considered the mild stress group, and plants irrigated at an interval of 9 days were labeled as being under severe stress. When subjecting plants to water deficient levels, they were treated with foliar application of ANE at rates of 0%, 0.1%, 0.2%, or 0.3%. Plants were protected from rainfall with a transparent 0.03 mm polythene sheet.

A preliminary experiment was conducted to determine the optimal drought levels before the starting the study. Moisture was measured with a moisture meter in order to ensure that the moisture level was uniform in all pots. In the mild stress group, irrigation was provided after the signs of incipient wilting appeared (6-day interval). In the severe stress group, irrigation was provided when the okra plants showed signs of temporary wilting (9-day interval), while plants irrigated after 3 days were kept as the control group.

ANE was brought from the market in liquid form. To prepare the 0.1% solution, 1 mL of ANE was dissolved in 999 mL of distilled water. To prepare the 0.2% solution, 2 mL of ANE was dissolved in 998 mL of distilled water. To prepare the 0.3% solution, 3 mL of ANE was dissolved in 997 mL of distilled water. The preparations were applied foliar with the help of a spray machine. Physiological parameters were measured 21 days after the application of the solution. Plants were treated with equal amounts of chemical with the help of the spray machine.

Pots were placed randomly using a CRD design with the following layout.


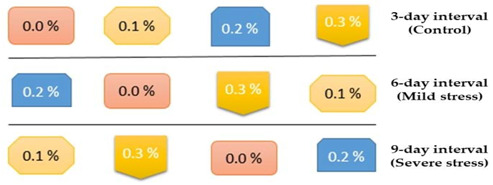


### 4.1. Chl a, Chl b, Total Chlorophyll, and Carotenoid

Chlorophyll was measured in accordance with Yang et al. [87]. The plant leaf samples (0.25 g) were weighed using a digital balance (Shimadzu, model AY220, Kyoto-Japan), dried in liquid nitrogen, and ground with a mortar and pestle. After grinding, the total pigments were extracted in 5 mL of 80% acetone using a 10 mL Pasteur pipette. After centrifugation (Centurion1020 D.E) of each sample at 1500× *g* for 5 min, a crude extract with a light green to green solution was obtained. The pellet was discarded, and the absorbance of the supernatant was measured using a 1 cm pathlength cuvette at 663.6, 646.6, and 440.5 nm on a UV–VIS spectrophotometer (GENESYS 10 UV–VIS, Thermo Spectronic, Rochester, New York, NY, USA). These wavelengths represent the absorption peaks of chlorophyll-a, chlorophyll-b, and carotenoids, respectively.

The chlorophyll-a, chlorophyll-b, total chlorophyll, and carotenoid contents were calculated using the following equations [88,89]:

Chlorophyll a (µg/mL) = 12.25A_663.6_ − 2.55A_646.6_ (1)

Chlorophyll b (µg/mL) = 20.31A646.6 − 4.91A663.6(2)

Total Chlorophyll (µg/mL) = 17.76A646.6 + 7.34A663.6(3)

Carotenoids (µg/mL) = 4.69A440.5–0.267 Chlorophyll a + Chlorophyll b.(4)

### 4.2. Proline Determination

The proline content was determined in accordance with Bates et al. [90]. Approximately 0.5 g of leaf tissue was homogenized in 10 mL of 30% sulfosalicylic acid and filtered using filter paper. Two milliliters of acid ninhydrin (1.25 g ninhydrin in 30 mL of glacial acetic acid) was added to the filtrate. The filtrate was mixed thoroughly and heated in a water bath at 100 °C for 1 h. The mixture was then cooled, and 4 mL of toluene was added and mixed. The chromophore containing toluene was separated from the aqueous phase, and its absorbance was read at 520 nm using a spectrophotometer (GENESYS 10 UV–VIS, Thermo-Spectronic, and Rochester, New York, NY, USA). The concentration was determined from a standard curve using the following equation:

µmole proline/g fresh weight = (µg proline/mL × mL of toluene/115.5)/g of sample

### 4.3. Antioxidant Enzymes

Five-tenths gram of healthy leaf samples was collected from all of the treatments in replicate and ground in liquid nitrogen. They were homogenized in phosphate buffer (0.05 mol/L phosphate buffer, pH 7.8) having 1% (*w*/*v*) polyvinylpyrrolidone (PVP). The solution was transferred to tubes and was centrifuged at 12,000× *g* at a temperature of 4 °C for 20 min. The supernatant obtained was used for assaying the activities of ascorbate peroxidase (APX;EC 1.11.1.11), peroxidase (POD; EC 1.11.1.7), and catalase (CAT; EC 1.11.1.6) [91].

The APX activity was measured using the method described by Asada et al. [92] with slight modifications. The reaction mixture, a total of 1 mL, contained 50 mM potassium phosphate buffer (pH, 7.0), 0.5 mL ascorbic acid, 0.1 mM H_2_O_2_, and 200 µL enzyme extract. The change in absorbance was recorded at 290 nm for 1 min using a spectrophotometer (GENESYS 10 UV–VIS, Thermo-Spectronic, and Rochester, New York, NY, USA). The enzyme activity was expressed as u per milligram of protein (where u is a 0.1 change in absorbance per minute per milligram of protein).

The activity levels of CAT and POD were determined in accordance with Chance and Maehly [93] with slight modifications. The reaction mixture for CAT was a total of 3 mL^−1^ containing 50 mM phosphate buffer (pH, 7.0), 5.9 mM H_2_O_2_, and 100 µL enzyme extract. The change in the absorbance of the solution was recorded every 20 s (caused by H_2_O_2_ decomposition in H_2_O) at 240 nm and expressed as micromoles of H_2_O_2_ decomposed per minute per milligram of protein. The reaction mixture for POD was a total of 3 mL containing 50 mM phosphate buffer (pH, 7.0), 20 mL guaiacol, 40 mM H_2_O_2_, and 1 µL of the enzyme extract. The change in the absorbance of the reaction solution was recorded every 20 s at 470 nm using a spectrophotometer (GENESYS 10 UV–VIS, Thermo Spectronic, Rochester, New York, NY, USA). One unit of POD is equivalent to the change in absorbance per milligram of protein.

### 4.4. Anthocyanin Content

The anthocyanin content was extracted and estimated using the method described by Zhang and Quantick [94]. In a mortar and pestle, 500 mg of the tissue was ground with 10 mL of 1% methanol, and this process was repeated three times. The homogenate was centrifuged at 19,000× *g* for 15 min. The resulting supernatant was diluted in 1% HCl–methanol to 50 mL. The absorption of diluents was measured at 530 nm using a spectrophotometer (GENESYS 10 UV–VIS, Thermo Spectronic, Rochester, New York, NY, USA), and the anthocyanin content was expressed per milligram of fresh weight.

### 4.5. Statistical Analysis

The data obtained were subjected to ANOVA appropriate for the CRD design with the Statistix 8.1 software package (Analytical Software Inc., Tallahassee, FL, USA). Significant (*p* < 0.05) results are expressed as the Tukey HSD in accordance with Steel and Torrey [95].

## 5. Conclusions

We conclude that foliar application of ANE minimizes the damaging effects of drought stress in okra plants by enhancing physiological and biochemical parameters such as the content of chlorophyll, carotenoid, and anthocyanin. ANE directly increases the proline content and antioxidant activity, increasing the ability of the plant to scavenge excess ROS produced under drought stress. Further mechanistic research is necessary to clarify the effects of foliar ANE on leaf anatomical structures and the pathway involved in improving the plant’s overall biochemical reaction under abiotic stress.

The effect of ANE was more prominent when given at a dose of 0.3%. Use of ANE is recommended to improve drought tolerance in okra.

## Figures and Tables

**Table 1:** Effects of *Ascophylum nodosum* extract on the chlorophyll-a (µg mL^−1^), chlorophyll-b (µg mL^−1^), total chlorophyll (µg mL^−1^), carotenoid (µg mL^−1^), and anthocyanin (mg g^−1^) contents of okra under water deficient conditions.

Treatments	Chlorophyll-a (µg mL^−1^)	Chlorophyll-b (µg mL^−1^)	Total Chlorophyll (µg mL^−1^)	Carotenoid (µg mL^−1^)	Anthocyanin (mg g^−1^)
ANE Levels (%)					
0	24.56 d	12.55 d	37.11 d	8.67 d	0.6479 d
1	26.32 c	13.23 c	39.55 c	9.50 c	0.6929 c
2	28.05 b	14.35 b	42.40 b	10.60 b	0.7121 b
3	30.61 a	16.35 a	46.96 a	12.40 a	0.7635 a
Tukey HSD	1.3196 *	0.6821 *	1.7369 *	0.4310 *	0.0216 *
Drought Stress (DS)					
Control	34.01 a	16.77 a	50.78 a	12.85 a	0.5235 c
Mild stress	25.64 b	13.67 b	39.31 b	9.85 b	0.7160 b
Severe stress	22.50 c	11.92 c	34.32 c	8.17 c	0.8728 a
Tukey HSD	1.0346 *	0.5348 *	1.3617 *	0.3379 *	0.0169 *
ANE × DS	2.9863 *	1.5435 *	3.9304 *	0.9752 *	0.0488 *

Means followed by the same letter in each column are not significantly different. * = significant at *p* < 0.05.

**Table 2:** Effects of *Ascophylum nodosum* extract on the proline (µmole g^−1^), APX (µ mg^−1^ protein), POD (µ mg^−1^ protein), and CAT (µ mg^−1^ protein) contents of okra under water deficient conditions.

Treatments	Proline (µmole g^−1^)	APX (µ mg^−1^ Protein)	POD (µ mg^−1^ Protein)	CAT (µ mg^−1^ Protein)
ANE Levels (%)				
0	20.99 d	16.87 d	24.29 d	19.52 d
1	22.81 c	17.81 c	25.51 c	20.45 c
2	23.58 b	18.83 b	26.95 b	21.77 b
3	25.65 a	19.79 a	28.34 a	22.89 a
Tukey HSD	0.8686 *	0.4857 *	0.6849 *	0.6565 *
Drought Stress(DS)				
Control	15.98 c	17.236 c	24.67 c	19.93 c
Mild stress	23.74 b	18.25 b	26.15 b	20.96 b
Severe stress	30.06 a	19.94 a	28.01 a	22.58 a
Tukey HSD	0.6810 *	0.3808 *	0.5370 *	0.5147 *
ANE × DS	1.9656 *	1.0990 *	1.5499 *	1.4857 *

Means followed by the same letter in each column are not significantly different. * = significant at *p* < 0.05.

## Data Availability

Most of the data are available in the manuscript.
